# Proliferation of Luteal Steroidogenic Cells in Cattle 

**DOI:** 10.1371/journal.pone.0084186

**Published:** 2013-12-27

**Authors:** Shin Yoshioka, Hironori Abe, Ryosuke Sakumoto, Kiyoshi Okuda

**Affiliations:** 1 Graduate School of Natural Science and Technology, Okayama University, Okayama, Japan; 2 Graduate School of Environmental and Life Science, Okayama University, Okayama, Japan; 3 Reproductive Biology Research Unit, National Institute of Agrobiological Sciences, Tsukuba, Japan; Baylor College of Medicine, United States of America

## Abstract

The rapid growth of the corpus luteum (CL) after ovulation is believed to be mainly due to an increase in the size of luteal cells (hypertrophy) rather than an increase in their number. However, the relationship between luteal growth and the proliferation of luteal steroidogenic cells (LSCs) is not fully understood. One goal of the present study was to determine whether LSCs proliferate during CL growth. A second goal was to determine whether luteinizing hormone (LH), which is known have roles in the proliferation and differentiation of follicular cells, also affects the proliferation of LSCs. Ki-67 (a cell proliferation marker) was expressed during the early, developing and mid luteal stages and some Ki-67-positive cells co-expressed HSD3B (a steroidogenic marker). DNA content in LSCs isolated from the developing CL increased much more rapidly (indicating rapid growth) than did DNA content in LSCs isolated from the mid CL. The cell cycle-progressive genes *CCND2* (cyclin D2) and *CCNE1* (cyclin E1) mRNA were expressed more strongly in the small luteal cells than in the large luteal cells. LH decreased the rate of increase of DNA in LSCs isolated from the mid luteal stage but not in LSCs from the developing stage. LH suppressed *CCND2* expression in LSCs from the mid luteal stage but not from the developing luteal stage. Furthermore, LH receptor (*LHCGR*) mRNA expression was higher at the mid luteal stage than at the developing luteal stage. The overall results suggest that the growth of the bovine CL is due to not only hypertrophy of LSCs but also an increase in their number, and that the proliferative ability of luteal steroidogenic cells decreases between the developing and mid luteal stages.

## Introduction

The corpus luteum (CL) is a transient endocrine gland formed from a ruptured follicle after ovulation. The CL produces progesterone (P4), which is required for the establishment and maintenance of pregnancy in mammals [1,2]. The CL is one of the fastest growing tissues in adult female mammals [3,4]. During the first 10 days of the 21-day bovine estrous cycle, luteal weight increases by 20- to 30-fold [4]. The CL reaches structural and functional maturity at the mid luteal stage (day 10) and then regresses at the end of the estrous cycle [5,6]. 

The CL mainly consists of (1) large luteal cells that are presumably derived from follicular granulosa cells, (2) small luteal cells that are presumably derived from follicular theca cells and (3) non-steroidogenic cells such as endothelial cells, fibroblasts and immune cells [7-10]. As the CL forms, it becomes highly vascularized and the vascularization is accompanied by a rapid proliferation of luteal endothelial cells [11-13]. During growth of the CL, luteal steroidogenic cells (LSCs) undergo dynamic changes [4,6], but there is no clear evidence that they proliferate. 

One approach to determining whether LSCs proliferate is to examine genes that regulate cell proliferation and cell differentiation. The regulation of both cell proliferation and differentiation is essential for normal organogenesis in order to obtain appropriate size, morphology and function [14]. For tissue homeostasis, differentiation is usually coordinated with the exit from the cell cycle [15]. The cell cycle is regulated by cyclin-dependent kinases (CDK) and several proteins that either stimulate or inhibit their activity [16]. Progression through the G1-phase is controlled mainly by CDK4/6 and CDK2 in association with CCND (cyclin D) and CCNE (cyclin E), respectively. Entry of cells into the S-phase involves the cooperation of CDK4/6 and D-type cyclins with CCNE-CDK2 complex [17,18]. In contrast to cyclins, CDK inhibitors such as CDKN1A (p21^Cip1^) and CDKN1B (p27^Kip1^) are negative regulators that arrest the cell cycle by binding to and inhibiting the activity of cyclin-CDK complexes [19]. However, it is not known to what extent genes that regulate cell proliferation are involved in controlling luteal growth.

Luteinizing hormone (LH) regulates a variety of ovarian functions. Activation of the LH receptor (LHCGR) in follicular cells by a preovulatory LH surge causes ovulation and rapidly initiates a program of terminal differentiation of the granulosa cells in a process termed luteinization [20]. Cessation of cell proliferation during luteinization is associated with a progressive loss of positive cell cycle regulators and with increased expression of the CDK inhibitors CDKN1A and CDKN1B [21,22]. LH also down-regulates *CCND2* mRNA and protein and induces CDK inhibitors in mice granulosa cells [23]. Thus, LH seems to play an important role in the proliferation and differentiation of follicular cells. However, the role of LH in the proliferation of LSCs is unclear. 

To elucidate whether LSCs proliferate during CL growth, we examined 1) the expression of KI-67, a cell proliferation marker, and HSD3B (also known as 3β-HSD), which is a marker specific for steroidogenic cells, in bovine luteal tissue, 2) the expression of cell cycle-related genes and PTEN (phosphatase and tensin homolog; a key regulator of cell proliferation) in freshly isolated LSCs and 3) the proliferation of cultured LSCs isolated from the developing and mid CL. To determine which cell types of LSCs proliferate, we compared cell cycle-related genes and *PTEN* mRNA levels between large and small luteal cells. We also examined the effects of LH on the proliferation of cultured LSCs and their expression of cell cycle-related genes.

## Materials and Methods

### Ethics Statement

In this study, we did not perform any animal experiments. The ovaries were collected from non-pregnant Holstein cows at a local abattoir (Tsuyama Meat Center) in accordance with protocols approved by local institutional animal care. All the samples and data analyzed in the present study were obtained with the permission of the above center.

### Collection of CL

The stages of the estrous cycle were identified by macroscopic observation of the ovary and uterus as described previously [24]. CL tissues were collected from cows at five different stages of the estrous cycle (early: Days 2-3; developing: Days 5-7; mid: Days 9-12; late: Days 15-17; regressed luteal stage: Days 19-21). For cell culture experiments, the ovaries with CL were submerged in ice-cold physiological saline and transported to the laboratory.

For immunohistochemistry, the CL tissues were immediately separated from the ovaries and dissected free of connective tissue. Tissue samples were fixed in 10% (v/v) neutral phosphate buffer formalin (pH 7.0) for 24 h at room temperature and then embedded in paraffin wax. The samples were cut into 4 &[mu]m sections and mounted onto glass microscope silanized slides (Dako).

### Cell isolation

Luteal tissue was enzymatically dissociated and luteal cells were cultured as described previously [25]. The luteal cells were suspended in a culture medium, DMEM, and Ham’s F-12 medium [1:1 (vol/vol); Sigma-Aldrich, Inc., St. Louis, MO; no. D8900] containing 5% calf serum (Life Technologies, Inc., Grand Island, NY; no. 16170-078) and 20 μg/ml gentamicin (Life Technologies; no. 15750-060). Cell viability was greater than 85% as assessed by trypan blue exclusion. The cells in the cell suspension consisted of about 75% small luteal cells, 20% large luteal cells, 5% endothelial cells or fibrocytes, and no erythrocytes. Some of the freshly isolated LSCs were washed with PBS (-) then used for gene analysis. Small and large luteal cells were separately collected from freshly isolated LSCs under a microscope. About 300 cells were collected for each cell type. Cell size was measured by ocular and stage micrometers. Small and large luteal cells were identified by their sizes: <20 μm and >35 μm, respectively.

To confirm the identities of the small and large luteal cells, we determined the expression levels of *oxytocin* mRNA, which is highly expressed in large luteal cells, and *LHCGR* mRNA, which is highly expressed in small luteal cells. These expressions were as expected.

### Cell culture

The dispersed luteal cells were seeded at 0.5 × 10^5^ viable cells per ml in 4-well cluster dishes (Nunc, Roskilde, Denmark; no. 176740) for cell proliferation assay or 2 x 10^5^ viable cells per ml in 24-well cluster dishes (Coster, Cambridge, USA; no. 3524) for gene analysis. They were then cultured in a humidified atmosphere of 5% CO_2_ in air at 37.5 C in a N_2_-O_2_-CO_2_-regulated incubator (ESPEC Corp., Osaka, Japan; no. BNP-110). After 16 h of culture, the medium was replaced DMEM/F-12 containing 5% calf serum (for cell proliferation assay) or DMEM/F-12 containing 0.1% [w/v] BSA, 5 ng/ml sodium selenite, 5 μg/ml transferring and 2 μg/ml insulin (for gene analysis) with or without LH (10 ng/ml). 

### Immunohistochemistry and immunofluorescence

Expression of KI-67, a cell proliferation marker, in bovine CL was demonstrated by immunohistochemistry. The 4 μm sections were deparaffined, rehydrated in a graded series of ethanol and washed in tap-water. Antigen retrieval was performed by placing the slides in boiling buffer (0.01 M citrate acid, pH 6) for 15 min. Endogenous peroxidase activity was quenched in methanol with 0.3% H_2_O_2_ for 10 min. Nonspecific binding was blocked at room temperature for 1 h in PBS-5% skim milk. Sections were incubated with an anti-mouse KI-67 antibody (1 : 200, Dako; M7240) overnight at 4 C. After incubation, the sections were washed with PBS and incubated with ImmPRESS UNIVERSAL reagent (Vector Laboratories; MP-7500) for 30 min according to the manufacturer’s instructions. The sections were visualized with 0.05% 3,3-diaminobenzidine tetrahydrochloride (DAB) in 0.01 M PBS (pH 7.4) containing 0.01% H_2_O_2_. 

Co-expression of KI-67 and HSD3B was detected by immunofluorescence. The sections were deparaffined, rehydrated in a graded series of ethanol and washed in tap-water. Antigen retrieval was performed by placing the slides in boiling buffer (0.01 M citrate acid, pH 6) for 15 min. Nonspecific binding was blocked at room temperature for 1 h in PBS-5% skim milk. Sections were incubated with the anti-mouse KI-67 (described above) and anti-goat HSD3B (1 : 100, Santa Cruz; sc-30820) overnight at 4 C. After incubation, the sections were washed with PBS three times followed by 1 h incubation with an appropriate secondary antibody diluted at 1 : 1000 (anti-mouse or goat IgG conjugated with Alexa 488 or 594) at room temperature in the dark. Coverslips were mounted on slides with ProLong Gold antifade reagent with DAPI (Invitrogen; P36935). 

### Cell proliferation assay

Cellular proliferation of bovine developing and mid luteal cells was demonstrated by spectrophotometric method of Labarca and Paigen [26]. Bovine luteal steroidogenic cells isolated from developing and mid stage CL were cultured for 1, 4, 7 and 10 days, and DNA contents were measured at the end of culture.

### RNA isolation and cDNA synthesis

Total RNA was prepared from bovine CL throughout estrous cycle and isolated LSCs using TRIZOL reagent according to the manufacturer’s directions (Invitrogen, Carlsbad, CA; no. 15596-026). A small amount of total RNA was prepared from separately collected small luteal cells and large luteal cells using PureLink RNA micro scale kit (Invitrogen; no. 12183016) according to the manufacturer’s directions. The total RNA was reverse transcribed using a ThermoScript RT-PCR system (Invitrogen; no. 11146-016).

### Real-time PCR

Gene expression was measured by real-time PCR using the MyiQ (Bio-Rad, Tokyo, Japan) and the iQ SYBR Green Supermix (Bio-Rad; No. 170-8880) starting with 1 ng of reverse-transcribed total RNA as described previously [27]. Standard curves of sample cDNA were generated serial dilutions (1:2 to 1:1000). The expression of *GAPDH* was used as an internal control. 20 bp primers with 50-60% GC-contents were synthesized (Table 1). The PCR contents were 95 C for 15 min, followed by 45 cycles of 94 C for 15 sec, 55 C for 30 sec and 72 C for 30 sec. Use of the QuantiTect SYBR Green PCR system at elevated temperatures resulted in reliable and sensitive quantification of the PCR products with high linearity. 

**Table 1:** Primers used in quantitative RT-PCR.

Gene	Primer	Sequence (5’-3’)	Accession no.	Product (bp)
CCND2	Forward	TTCAGCAGGATGAGGATGTG	NM001076372	146
	Reverse	ACGGTACTGCTGCAGGCTAT		
CCNE1	Forward	TTCCTGCTGAAGATGCACAC	NM001192776	78
	Reverse	CTTTCTTTGCTTGGGCTTTG		
CDKN1A	Forward	AGCATCCTTTGCTTTCCTCA	NM001098598	72
	Reverse	AGAGAGTGCCACCCAGAGAA		
CDKN1B	Forward	AGATGTCAAACGTGCGAGTG	NM001100346	104
	Reverse	GCCAAAGAGGTTTCTGCAAG		
PTEN	Forward	TGACTGCTCCATCTCCTGTG	XM002698370	119
	Reverse	CCACAGCAGGTACACGATTG		
LHCGR	Forward	CTAGCCATCACGGGAAATGT	U20504	102
	Reverse	GTCTGCAAAGGAGAGGTTGC		
Oxytocin	Forward	CTTCTCCCAGCACTGAGACC	M25648.1	103
	Reverse	CCGGCTTTATTTCATTGTCA		

### Statistical analysis

All experimental data are shown as the mean ± sem. The statistical significance of differences in the contents of DNA and protein throughout the estrous cycle and the cellular proliferation of bovine mid luteal cells were assessed by ANOVA followed by a Fisher protected least significant difference procedure (PLSD) as a multiple comparison test. 

## Results

### Luteal weight, DNA and protein contents of bovine CL throughout the estrous cycle

The CL weight and protein contents (an index of cell size) increased from the early to late luteal stages and then decreased toward the regressed luteal stage, whereas DNA contents (an index of cell number) only increased from the early to developing luteal stages (Table 2).

**Table 2:** Luteal weight, DNA and protein contents of bovine CL throughout the estrous cycle.

	Early	Developing	Mid	Late	Regressed
Wet tissue weight	0.86±0.15^a^	2.29±0.20^b^	3.57±0.20^c^	4.25±0.88^c^	0.84±0.06^a^
(g/CL)					
DNA contents (mg/CL)	0.52±0.09^a^	2.00±0.55^bc^	2.21±0.18^c^	2.88±0.32^c^	1.10±0.27^b^
(Index of hyperplasia)					
Protein contents	55.5±28.4^a^	246.7±50.9^b^	396.8±13.2^c^	468.1±70.5^c^	71.3±7.2^a^
(mg/CL)					

### KI-67 expression in the CL throughout the estrous cycle

**Figure 1:**
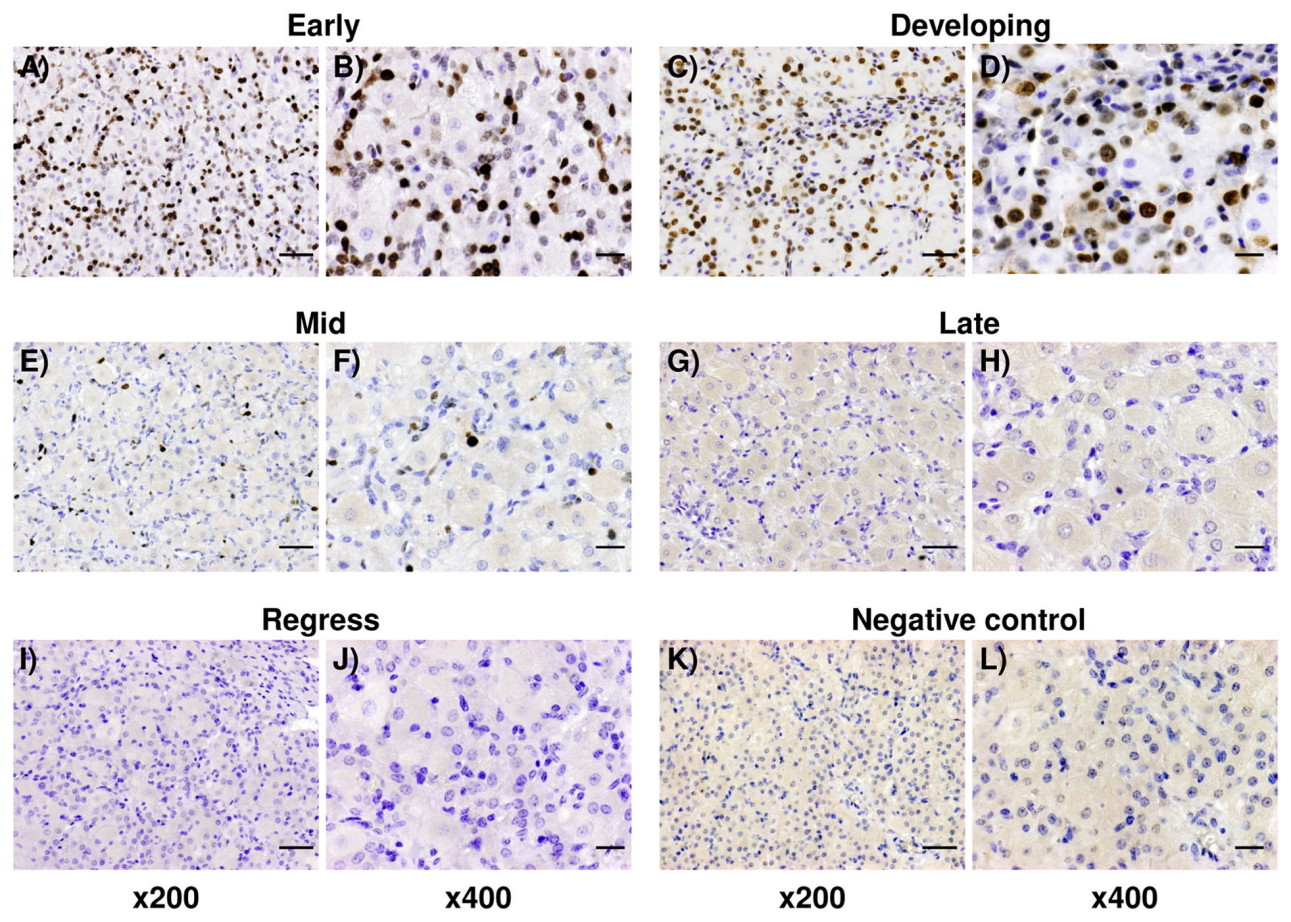
KI-67 expression in the bovine CL. Representative images of immunohistochemical staining for KI-67 in the bovine CL throughout the estrous cycle (A, B; early, C, D; developing, E, F; mid, G, H; late, I, J; regressed luteal stages). Scale bars represent 100 μm (low magnitude) and 50 μm (high magnitude).

KI-67-positive cells were localized in the CL from the early, developing and mid luteal stages and the rate of KI-67 staining was greater in the early and developing CL than in the mid CL (Figure 1A-F). In contrast, only a few KI-67-positive cells were localized in the late CL and no mitotic activity was observed in the regressed CL (Figure 1G-J).

### Double immunofluorescent staining of KI-67 and HSD3B

**Figure 2:**
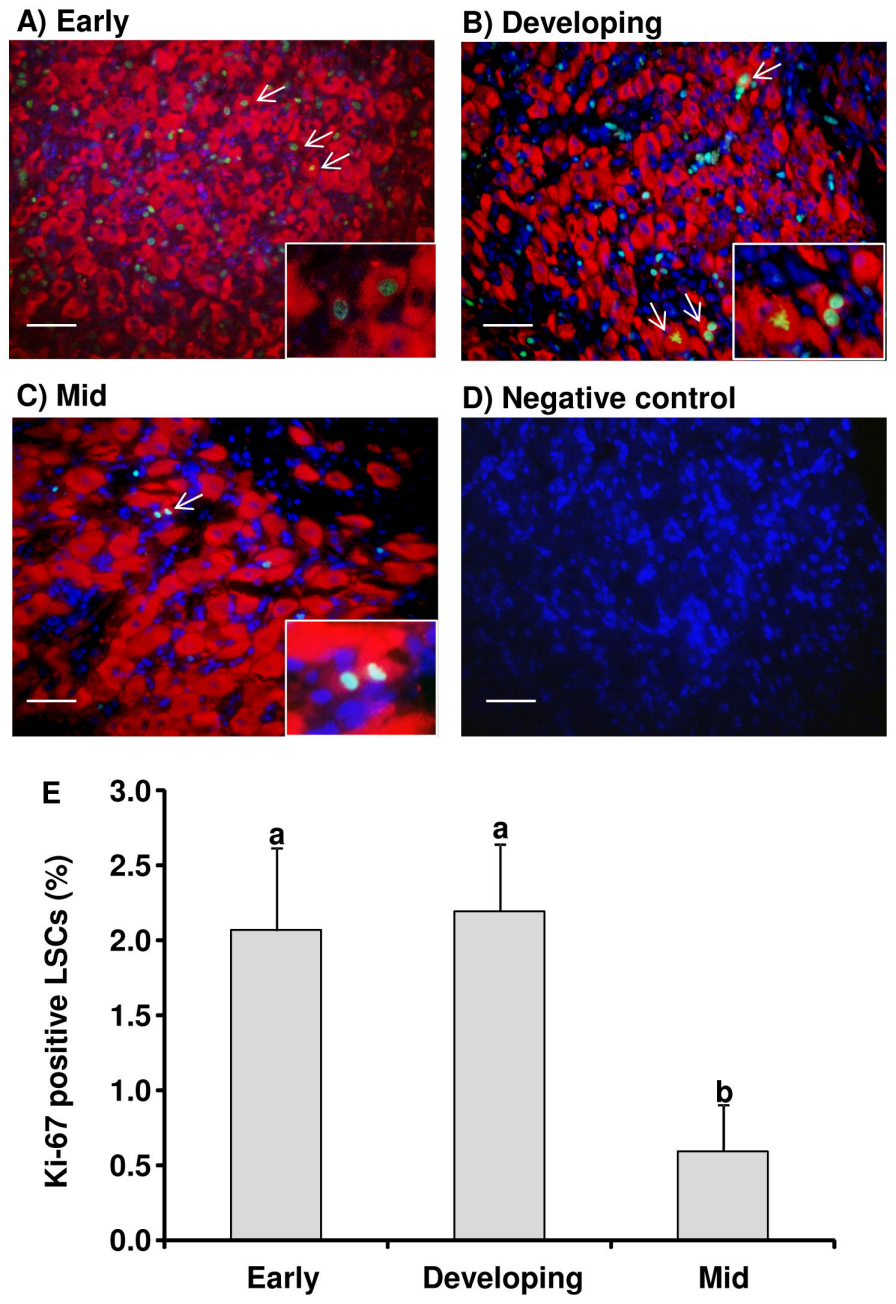
KI-67 and HSD3B expression in the bovine CL. Representative images of double-immunohistochemical staining for KI-67 and HSD3B in early (A), developing (B) and mid (C) bovine CL. Normal horse serum was used as a negative control (D). Scale bars represent 50 μm. (E) Percentage of KI-67 positive LSCs in the bovine CL (mean±SEM, n=3 experiments). Percentages in each luteal stage were derived from dividing the number of Ki-67 and HSD3B positive cells by total number of HSD3B positive cells. Cells were counted in three randomly chosen areas. Different superscript letters indicate significant differences (P<0.05).

The steroidogenic enzyme HSD3B was present in the CL tissues at the early, developing and mid stages. About 2% of KI-67 positive cells that also expressed HSD3B in the early and developing CL (Figure 2A, B, D) and less than 1% of cells co-expressed both cell markers in the mid CL (Figure 2C, D).

### Expression of cell cycle-related genes and PTEN in bovine freshly isolated LSCs

**Figure 3:**
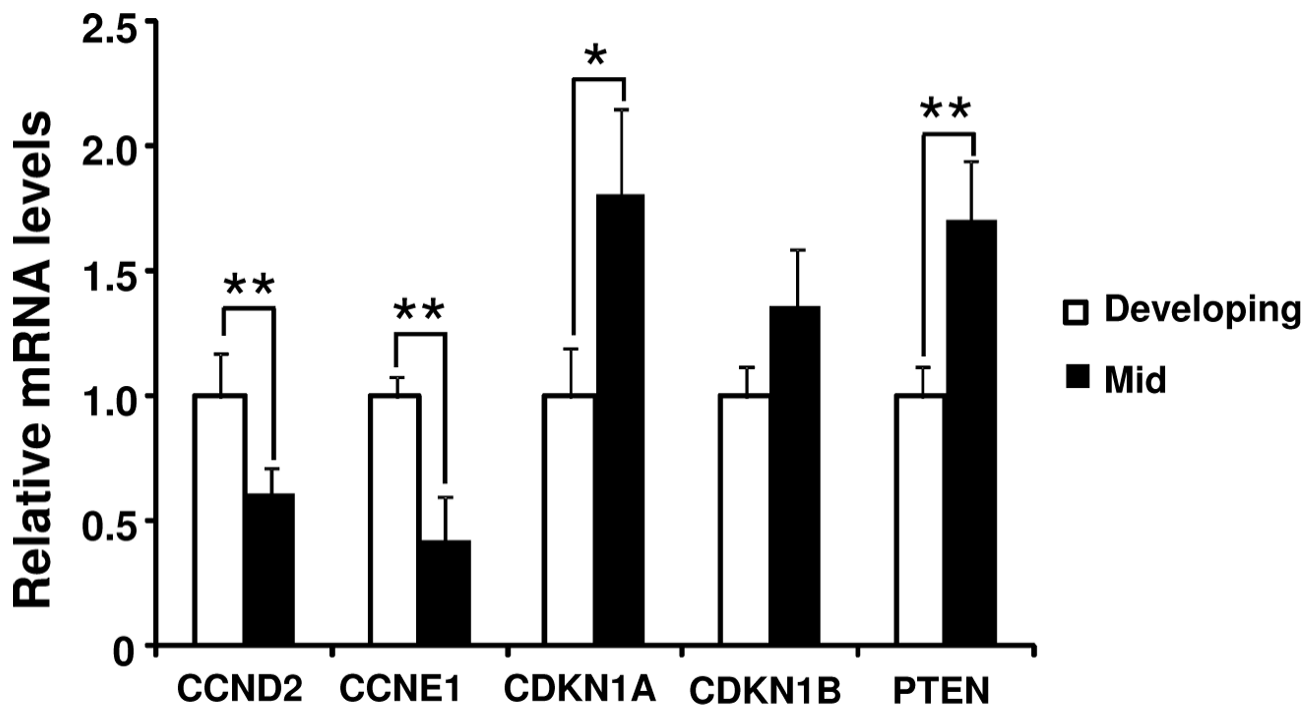
Cell cycle-related genes and *PTEN* expression in bovine freshly isolated LSCs. Fold differences of cell cycle-related genes such as *CCND2*, *CCNE1*, *CDKN1A*, *CDKN1B* and tumor suppressor gene *PTEN* mRNA between freshly isolated developing LSCs and mid LSCs. Single and double *Asterisks* indicate significant differences (* ; P<0.1, ** ; P<0.05), as determined by ANOVA followed by Fisher’s PLSD as a multiple comparison test.

*CCND2* and *CCNE1* mRNA were expressed more strongly in the LSCs from developing CL than in the LSCs from mid CL. On the other hand, *CDKN1A* tended to be expressed more strongly in the LSCs from mid CL than in the LSCs from developing CL. Furthermore, *PTEN* was expressed more strongly in the LSCs from mid CL than in the LSCs from developing CL (Figure 3). 

### Expression of cell cycle-related genes in bovine freshly isolated small and large luteal cells

**Figure 4:**
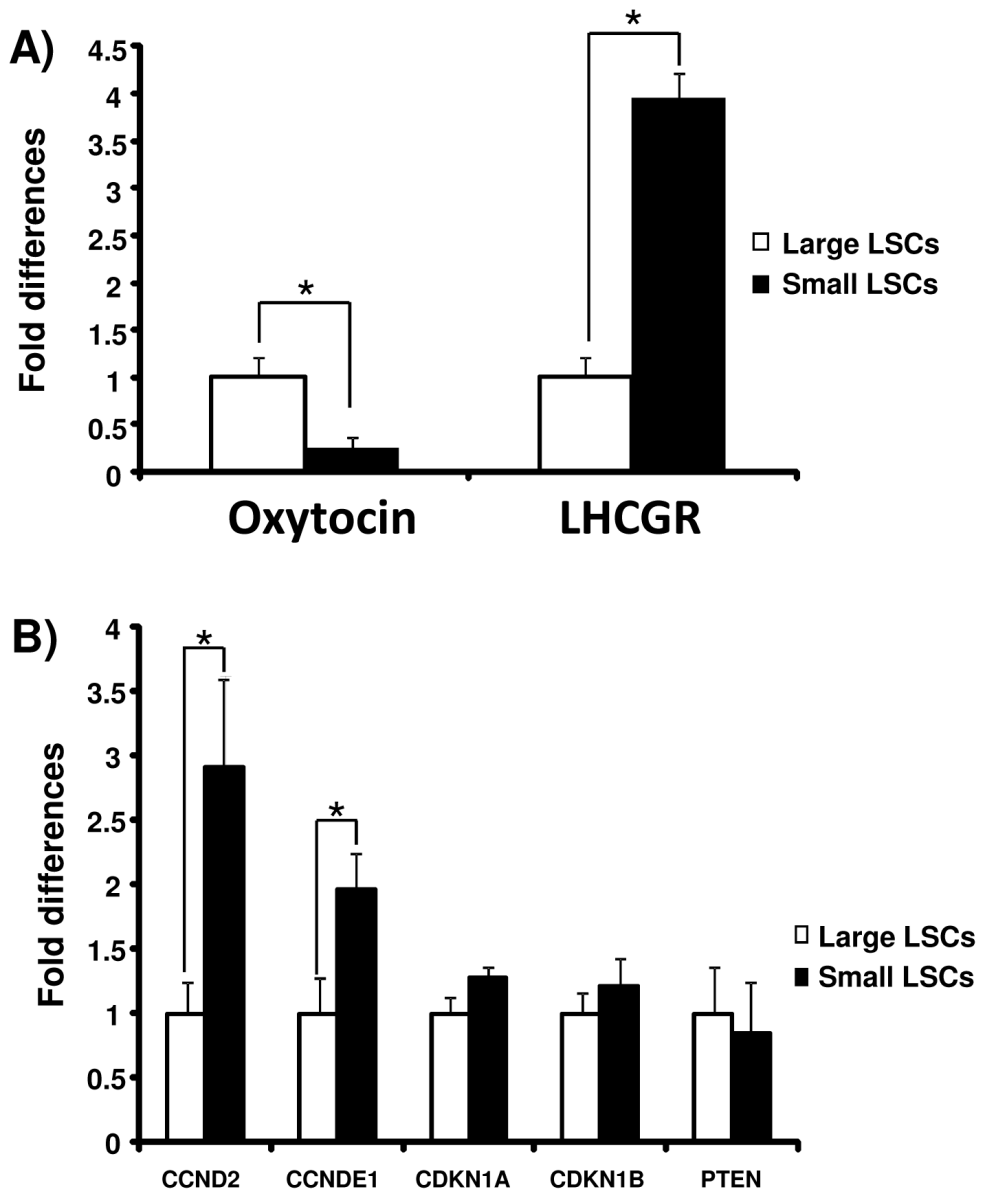
Cell cycle-related genes and *PTEN* expression in small and large luteal cells. Fold differences of oxytocin, LHCGR and cell cycle-related genes such as *CCND2*, *CCNE1*, *CDKN1A*, *CDKN1B* and tumor suppressor gene *PTEN* mRNA between large luteal cells and small luteal cells isolated from developing and mid luteal stages. *Asterisks* indicate significant differences (* P<0.05), as determined by ANOVA followed by Fisher’s PLSD as a multiple comparison test.

The identities of small and large luteal cells, were confirmed by the expressions of *oxytocin* mRNA and *LHCGR* mRNA, respectively (Figure 4A). The cell cycle-progressive genes *CCND2* and *CCNE1* mRNA were expressed more strongly in the small luteal cells than in the large luteal cells (Figure 4B).

### Cellular proliferation of cultured bovine LSCs

**Figure 5:**
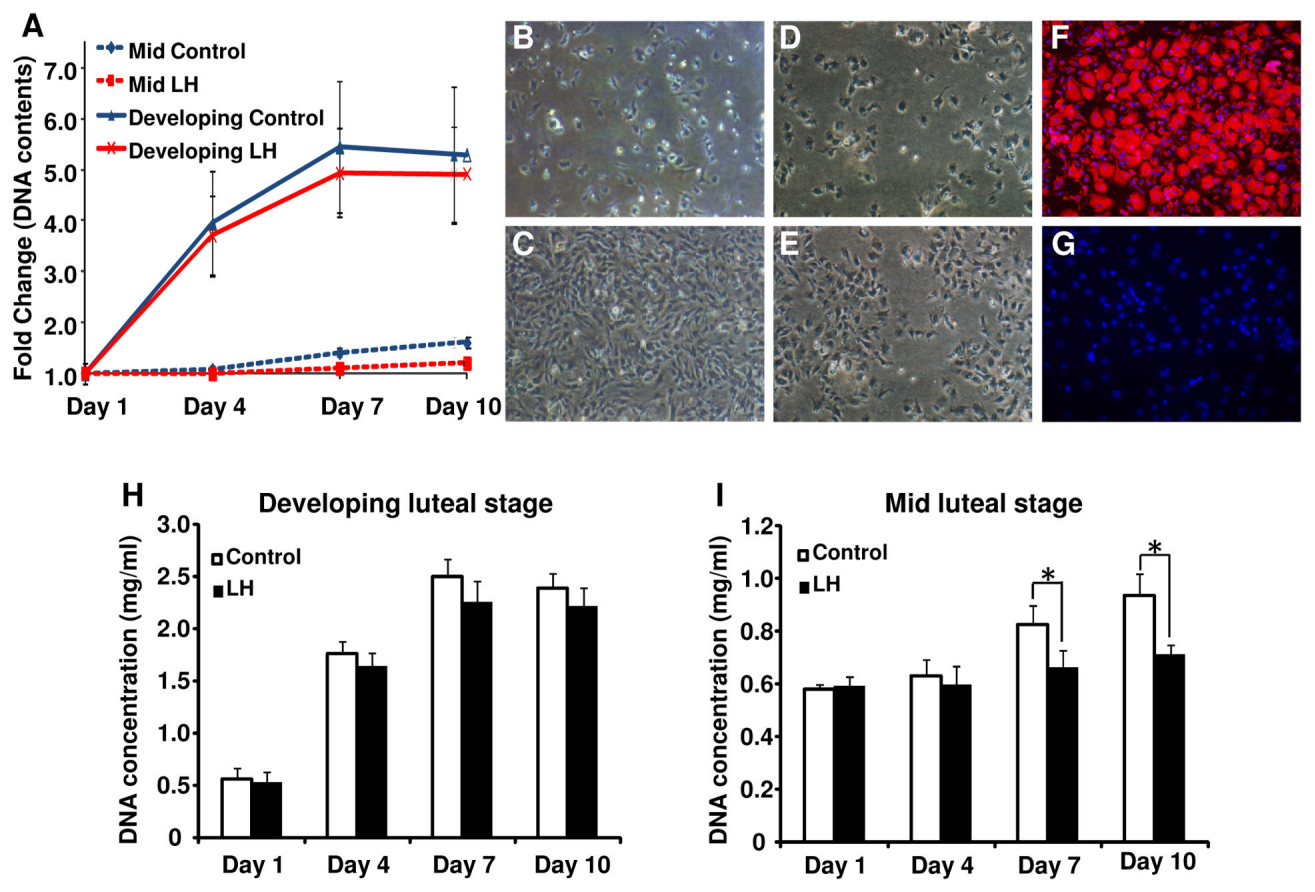
Proliferation of cultured LSCs. (A) Cellular proliferation was determined by DNA assay at the indicated times with or without LH (10 ng/ml) after the LSCs were seeded (n=5). Daily observation of luteal steroidogenic cells at (B) 1, (C) 4 days from developing CL and (D) 1, (E) 4 days from mid CL were shown. To confirm that cultured cells were steroidogenic cells, the LSCs isolated from mid luteal stage were cultured for 24 h, then immunohistochemical staining with HSD3B was performed (F). PBS was used as a negative control (G). (H) and (I) show the effect of LH (10 ng/ml) on cellular proliferation of bovine luteal cells isolated from the developing and mid CL, respectively (mean±SEM, n-5 experiments). *Asterisks* indicate that LH had a significant effect on DNA concentration (P<0.05), as determined by ANOVA followed by Fisher’s PLSD as a multiple comparison test.

DNA contents were used as an index of cell proliferation. Liner increases in DNA contents of cultured LSCs were observed in control and LH-treated cells isolated from both the developing and mid luteal stages, although the DNA contents of the mid luteal stage were less than those of the developing stage (Figure 5A). LH decreased the DNA contents in bovine LSCs obtained from the mid luteal stage but not from the developing luteal stage (Figure 5H, I). The LSCs increased after they were seeded (Figure 5B-E). More than 95% of the cells positively immunostained with HSD3B (Figure 5F).

### Effect of LH on cell cycle-related genes and PTEN in bovine LSCs

**Figure 6:**
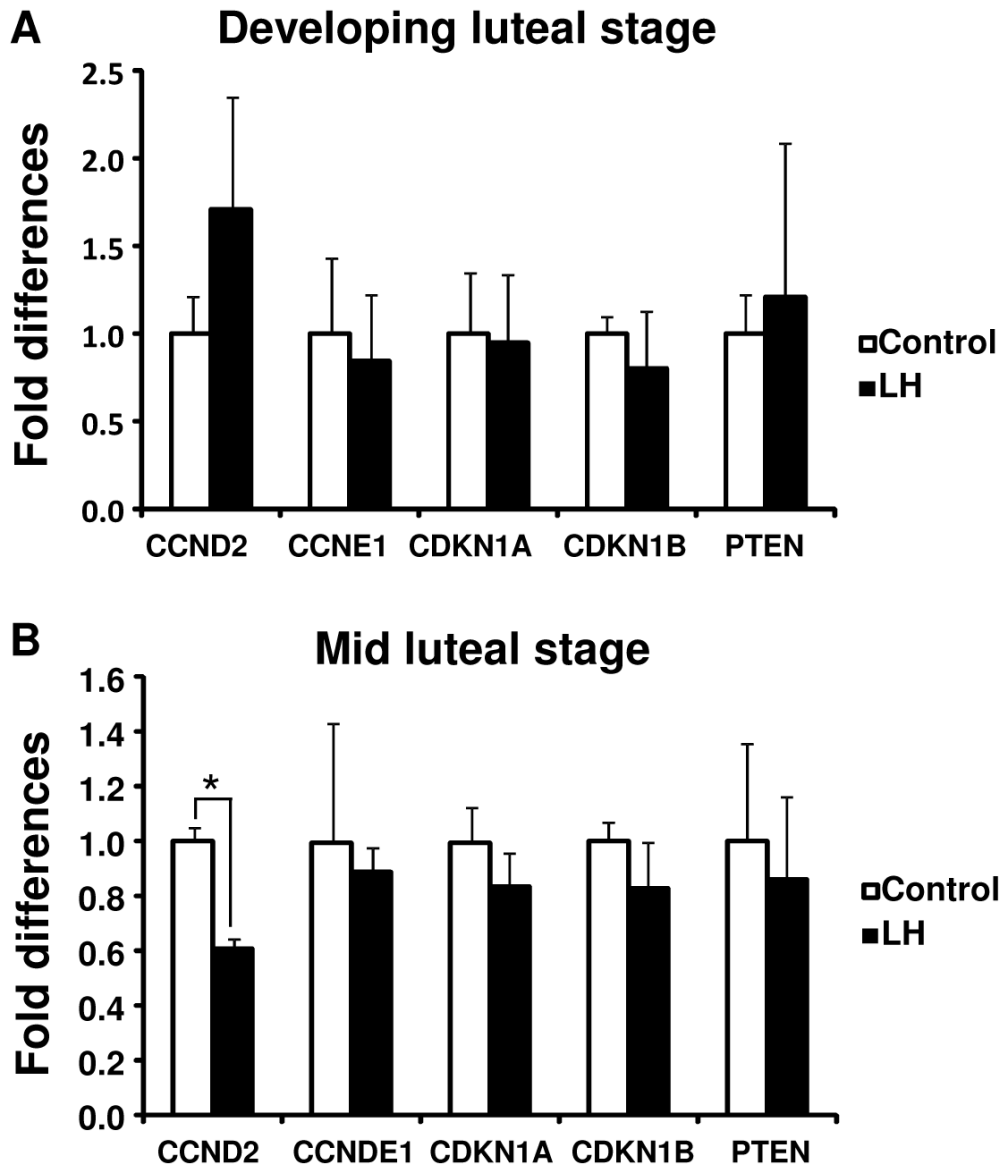
Effect of LH on cell cycle-related genes and *PTEN* expression in cultured LSCs. *CCND2*, *CCNE1*, *CDKN1A*, *CDKN1B* and *PTEN* mRNA in cultured LSCs isolated from the developing (A) and mid (B) CL (mean±SEM, n=4 experiments). The cells were exposed to LH (10 ng/ml) for 24 h. Asterisks indicate significant differences (P<0.05), as determined by ANOVA followed by Fisher’s PLSD as a multiple comparison test.

LH decreased *CCND2* mRNA level in LSCs isolated from at the mid luteal stage, but not in LSCs isolated from the developing luteal stage. LH did not affect other LSC-expressed genes such as *CCNE1*, *CDKN1A*, *CDKN1B* and *PTEN* in either the developing or mid LSCs (Figure 6). 

### LHCGR mRNA expression in the CL throughout the estrous cycle

**Figure 7:**
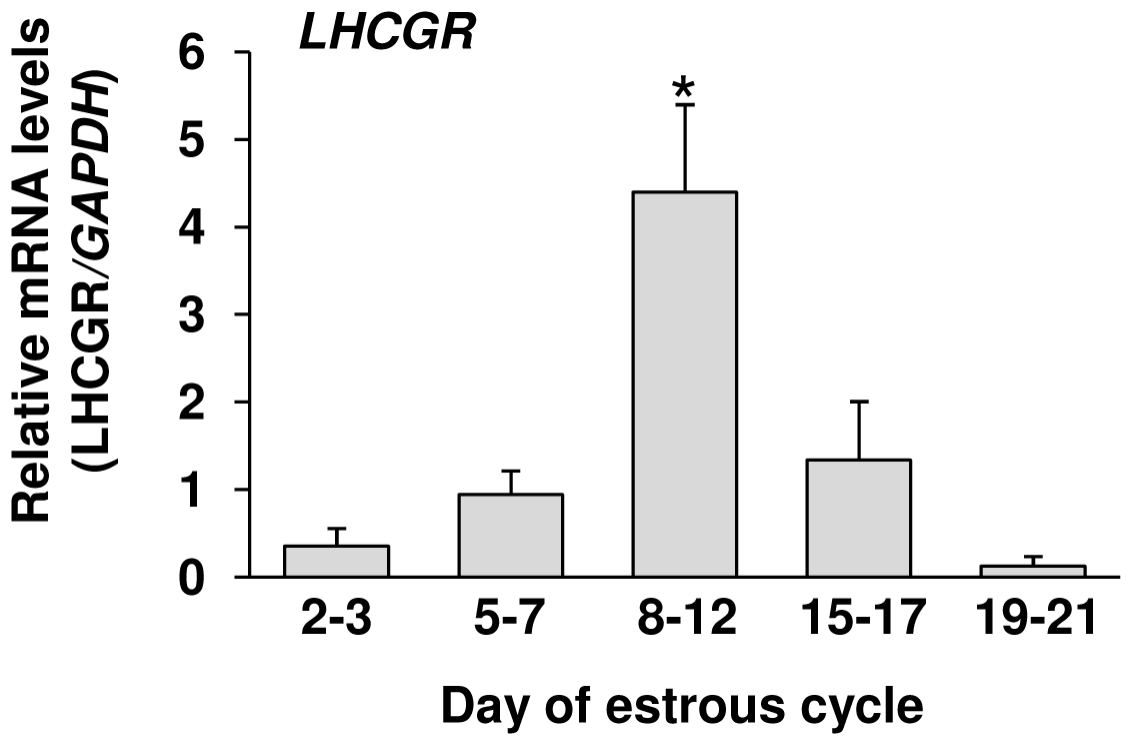
LHCGR expression in the bovine CL. Relative levels of *LHCGR* mRNA in bovine CL throughout the estrous cycle (n=4). *Asterisks* indicate significant differences (P<0.05), as determined by ANOVA followed by Fisher’s PLSD as a multiple comparison test.

*LHCGR* mRNA was expressed in bovine CL throughout the estrous cycle, and was significantly higher (P<0.05) at the mid luteal stage than at the other luteal stages (Figure 7). 

## Discussion

While the corpus luteum (CL) has a central role in establishing and maintaining pregnancy, the mechanisms of CL growth are not completely understood. Although cells positive for KI-67 (a cell proliferation marker) have been reported in the growing CL [4,28,29], there are still opposite opinion in this matter because of lacking in evidence [6]. The present results clearly show that bovine LSCs proliferate during CL growth. First, we observed cells that were positive for both HSD3B, a steroidogenic marker, and KI-67 in the early, developing and mid CL (Figure 2). Second, cell cycle progressive genes such as *CCND2* and *CCNE1* were expressed more strongly in LSCs obtained from the developing CL than in LSCs obtained from the mid CL (Figure 3). Third, cultured bovine LSCs isolated from the bovine CL proliferated until they reached confluence (Figure 5). 

To confirm that LSCs proliferate during CL growth, we first investigated the DNA and protein contents in luteal tissues that provide a good index of luteal growth, since both the DNA and protein contents have been correlated with luteal weight [4,29]. Although CL weight and protein contents increased until the mid luteal stage, DNA contents stopped increasing at the developing luteal stage (Table 2). Double-immunostaining for HSD3B and KI-67 provided more direct evidence for the proliferation of LSCs during CL growth. HSD3B and Ki-67 co-positive cells were more abundant at the developing luteal stage than at the mid luteal stage (Figure 2). These results are consistent with those of previous studies [4,28,29]. In addition, cell cycle progressive genes such as *CCND2* and *CCNE1* mRNA were expressed more strongly in the LSCs taken from the developing CL (Figure 3) and LSCs from the developing CL proliferated faster than those from the mid CL (Figure 5A). Luteal growth is generally thought to depend on hypertrophy rather than hyperplasia [30-32]. However, the results of the previous and present studies suggest that bovine luteal growth is due to both an increase in cell number and hypertrophy during the early and developing luteal stages.

The main difference between large and small luteal cells in bovine CL is their diameters [33-36]. The smallest diameter of large luteal cells is 25 μm [37] and the average diameters of the large and small luteal cells were 38.4 μm and 17.2 μm, respectively [9]. The large luteal cells and the small luteal cells are believed to be derived from follicular granulosa cells and follicle theca cells, respectively [5,38]. It has been reported that the cell cycle of granulosa cells is arrested after the ovulatory stimulus in mouse [23], rat [39] and rhesus macaques [40]. The theca cells were found to proliferate faster than granulosa cells after inducing in vitro luteinization by 5% fetal bovine serum　[41]. Because the size of the KI-67-positive steroidogenic cells were less than 20 μm in the present study, the proliferating LSCs seem to be mostly small luteal cells. Furthermore, cell proliferation progressive genes were expressed more strongly in the small luteal cells than in the large luteal cells (Figure 4). Thus, the present findings also support the idea that only small luteal cells proliferate during luteal development. The differences between the proliferation of large and small luteal cells suggest that the mechanisms of terminal differentiation are distinct between these two types of cells.

In contrast to the early and developing luteal stages, the mid luteal stage was characterized by relatively few KI-67-positive LSCs (Figure 2) and the rate of increase of DNA contents was slower in LSCs isolated from the mid CL than in LSCs isolated from the developing CL (Figure 5A). In addition, cell cycle progressing genes (CCND2 and CCNE1) were expressed more strongly at the developing luteal stage than at the mid luteal stage (Figure 3). These findings indicate that the terminal differentiation of bovine luteal cells occurs between the developing and mid luteal stages. Since LH induces the differentiation of proliferative follicular cells to non-proliferative luteal cells [21,22], LH is one of the possible substances controlling cellular proliferation of LSCs. In the present study, LH treatment decreased the proliferation of cultured bovine LSCs isolated from the mid CL coincident with down-regulating the *CCND2* level (Figure 5I and Figure 6B). This result suggests that the cellular proliferation of LSCs is down-regulated by LH. Furthermore, *LHCGR* mRNA expression was higher at the mid luteal stage than at the other luteal stage (Figure 7). Based on the above findings, the increased LHCGR and its activation by LH decrease the cellular proliferation of LSCs and assist the terminal differentiation of bovine LSCs. Further studies are needed to clarify the relationship between the LH receptor expression and luteal steroidogenic cells proliferation.

The PI3K/AKT pathway induces cell proliferation by modulating the expressions of cell cycle-related genes [42] and PTEN is a negative regulator of the PI3K/AKT pathway [43]. Thus, PTEN is one of the key regulators of cell proliferation. Our finding that *PTEN* mRNA level was higher in the freshly isolated LSCs obtained from developing stage CL than that from mid luteal stage CL (Figure 3) suggests that the PTEN expression level changes dramatically between the developing and mid luteal stages. While cellular proliferation of LSCs was down-regulated by LH, LH did not affect the *PTEN* mRNA expression in the present study. Recently, PTEN expression levels have been shown to be tightly controlled by biologically active RNAs such as miR-106-25 microRNA [44]. For example, miR-25 strongly down-regulates PTEN expression in melanoma cells [45]. Furthermore, miR-25 was expressed more strongly at the early luteal stage CL than at the late luteal stage CL in sheep [46]. These findings suggest that miR-25 is the one of the key regulators of PTEN. Further studies are needed to clarify the exact mechanisms regulating PTEN expression as well as cell cycle-related genes in bovine CL.

In conclusion, our results indicate that luteal growth depends not only on hypertrophy of LSCs but also on an increase in the number of small luteal cells, and suggest that the proliferative ability of luteal steroidogenic cells changes between the developing and mid luteal stages.
